# The Tubo‐ovarian abscess study (TOAST): A single‐center retrospective review of predictors of failed medical management

**DOI:** 10.1002/ijgo.70100

**Published:** 2025-03-31

**Authors:** Anna Marshall, Jordon Wimsett, Charlotte Handforth, Louise Unsworth, Jessica Wilson, Anna‐Marie Van Der Merwe, Charlotte Oyston

**Affiliations:** ^1^ Department of Obstetrics & Gynaecology Te Whatu Ora, Counties Manukau Auckland New Zealand; ^2^ Department of Obstetrics & Gynaecology University of Auckland Auckland New Zealand

**Keywords:** outcomes, pelvic inflammatory disease, surgical drainage, tubo‐ovarian abscess

## Abstract

**Objective:**

Tubo‐ovarian abscesses (TOAs) cause significant morbidity. Surgical intervention is required if broad‐spectrum intravenous antibiotics are unsuccessful. This study aimed to describe admission characteristics that predict failed medical management and to evaluate a previously developed risk score for predicting the need for surgical intervention in cases of TOA.

**Design:**

Single centre, retrospective cohort study.

**Setting and Patients:**

Patients admitted to a tertiary‐level public teaching hospital with a radiologically or surgically proven TOA between January 1, 2012 and December 31, 2018.

**Measures:**

Demographic and clinical details were obtained from electronic clinical records. Medical treatment was considered “failed” when surgical intervention was required beyond 24 h of antibiotics. Multivariable analyses using logistic regression was used to determine predictors of failed medical management. Risk scores were calculated as per Fouks et al. and a receiver operating characteristic curve was constructed to assess correlation with outcomes.

**Results:**

There were 425 patients and 522 admissions with TOA. In the first 24 h, 14% (72/522) of admissions were treated with a surgical intervention in addition to intravenous (IV) antibiotics, while 86% (450/522) were treated with IV antibiotics alone. In those treated with IV antibiotics alone, medical treatment was successful in 65% (293/450) of cases, with 35% (159/450) requiring additional surgical or radiological intervention prior to discharge.

Variables independently associated with failed medical treatment were fever at admission (adjusted odds ratio [aOR] 1.72, 95% confidence interval [CI] 1.11–2.67), larger mean diameter of TOA (2% higher odds for every 1‐mm increase in abscess size) and higher C‐reactive protein value (1% higher odds for every unit increase) at admission. The area under the curve (95% CI) for Fouks et al. scoring system was 0.63 (0.58–0.68), indicating poor discriminatory ability.

**Conclusions:**

A third of TOAs managed medically required surgical intervention. Fever, higher inflammatory markers, and larger mass were predictive of requiring surgery. However, a scoring system using these variables had poor discriminatory ability to predict treatment failure. Prospective studies are needed to determine whether earlier recourse to surgery can improve outcomes.

## 1 INTRODUCTION

A tubo‐ovarian abscess (TOA) is an inflammatory mass involving the fallopian tube and ovary, usually resulting from infection ascending from the lower genital tract.^1^ Abscess rupture can result in life‐threatening peritonitis, and long‐term consequences include subfertility, ectopic pregnancy, and chronic pain.^1^, ^2^, ^3^ Prompt identification and treatment of affected individuals has the potential to reduce these chronic sequalae. First‐line management of TOA is with broad‐spectrum intravenous (IV) antibiotics.^4^, ^5^ However, at least 30% of patients may require further intervention^3^, ^6^, ^7^ via guided drainage or surgery. Identifying those at high risk of antibiotic treatment failure and providing an early surgical intervention could reduce the length of hospitalization, the duration and severity of illness, hasten recovery, and reduce medical costs.^3^ However, removal or drainage also exposes patients to anesthetic or surgical complications, when a significant proportion of patients can be successfully managed with IV antibiotics alone.

Retrospective cohorts have described characteristics associated with treatment failure, including age, comorbidities, and laboratory and radiology markers.^2^, ^3^, ^6^, ^7^, ^8^, ^9^, ^10^, ^11^, ^12^, ^13^ In 2019, Fouks et al. published a clinical risk score for predicting the failure of conservative treatment in those with TOA. The risk scoring system is based on data from their local population, and uses age, white cell count, abscess diameter, and bilaterality. Our tertiary‐level public hospital serves a very different demographic to that described by Fouks et al., being ethnically diverse with a large Pacific population, and high rates of socioeconomic deprivation, obesity, and diabetes.^14^ Anecdotally, rates of TOA in our unit are high. We were therefore interested in reviewing outcomes related to TOA in our unit and evaluating how the score system developed by Fouks et al. performed. If this score system performed well, it would support wider use to guide decision‐making on need for further treatment.

The aim of this study was to determine the clinical characteristics associated with medical treatment failure in our unit. Our secondary aim was to evaluate the use of the score system developed by Fouks et al. to predict those who required surgical intervention in our population.

## 2 METHODS AND MATERIALS

The study was registered with and had full approval from the institution it was conducted within (Counties Manukau Health CMH registration no. 932). Due to the observational nature of the study, no further ethical approvals were required.

This was a single‐center retrospective cohort study. We included admissions for non‐pregnant persons admitted between January 1, 2012 and December 31, 2018 with a radiologically or surgically proven TOA.^1^ TOAs were radiologically proven where a computed tomography or ultrasound scan reported by a specialist radiologist described an adnexal mass consistent with or suspicious for a TOA. TOAs were considered surgically proven where an inflammatory mass involving the Fallopian tube and/or ovary was described at surgery. We excluded those with a pelvic mass thought to be due to non‐infective pathology (e.g., uncomplicated endometrioma), and those with uncomplicated pelvic inflammatory disease (PID).

### 2.1 Setting

Our institution provides 24‐h access to operating theaters and interventional radiology. All patients with suspected TOA receive IV ceftriaxone 2 g daily until apyrexial for 48 h, twice‐daily oral doxycycline 100 mg and metronidazole 400 mg for 14 days. IV gentamicin is added if no improvement occurs within 24–48 h. Patients are reviewed daily by a consultant gynecologist. There is no formal guidance about when intervention should be performed, but standard practice is for drainage to be arranged after 24–48 h of medical therapy if there is no improvement or if there is clinical deterioration. Surgical intervention involves drainage of abscess, removal of adnexa (adnexectomy), or pelvic clearance via either laparoscopic or open procedure. Radiological intervention involves drainage of the TOA via interventional radiology.

### 2.2 Data collection

A waiver of consent was obtained to review clinical information without obtaining individual informed consent. Potential cases were obtained through a search of clinical codes (tubo‐ovarian abscess, tuboovarian abscess, tubo ovarian abscess, TOA, pelvic abscess, pyosalpinx, pelvic collection, infected endometrioma, acute salpingitis) to identify admissions with TOA. Case note review was performed to ensure inclusion criteria were met and to obtain demographics (age, ethnicity), medical history (BMI [calculated as weight in kilograms divided by the square of height in meters], smoking status, presence of diabetes, recent surgery, presence of intrauterine contraceptive device (IUCD), prior history of PID, sexually transmitted infection [STI] or TOA), presenting signs (pyrexia with temperature of ≥37.5°C), admission investigations, imaging (modality, abscess measurements, uni/bilaterality) and admission/readmission information. Surgical details collected include approach (open or laparoscopic), drainage versus tissue removal, and complications.

Initial treatment was classified as either “medical management,” or “early surgical or early radiological intervention” when further intervention was planned within the first 24 h of admission in addition to medical therapy. For all medical management cases, we then compared admission characteristics and outcomes for those who improved with subsequent discharge from hospital without further intervention (“successful medical”) with those where a decision was made for further surgical intervention or radiological drainage more than 24 h after admission (“failed medical”).

### 2.3 Data analysis

Data management and analysis were undertaken using Stata v9.4 (SAS Institute, Cary, NC, USA). Categorical data were expressed as number and percentage. Continuous data were assessed for normality by visual inspection of histograms, and calculation of skewness and kurtosis scores. As data were not normally distributed, these were expressed as median (interquartile range [IQR]). Multivariable analyses were performed using logistic regression to examine predictors of failed medical treatment. Variables were chosen a priori, including age, ethnicity, presence of diabetes, fever ≥37.5°C on admission, bilateral abscess, largest abscess diameter, previous STI, PID, or TOA, intrauterine device in situ, and laboratory values (white cell count [WCC], C‐reactive protein [CRP], creatinine). A *P*‐value <0.05 was considered statistically significant. Missing data were rare, as reported in the results tables. Missing data were not imputed.

### 2.4 Validation of risk score

The risk assessment score developed by Fouks et al. was calculated for each admission where medical management was intended to be the primary means of treatment (i.e., no intervention was planned within the first 24 h of admission in addition to medical therapy).^1^ The risk score ranges from 0 to 5 and is calculated as follows: risk score = {[1 × age (>36 years)] + [1 × mean WCC (≥16 × 1000/mm^3^)] + [2 × abscess diameter (≥70 mm)] + [1 × bilateral abscess]}. The intervention rates calculated by Fouks et al. for those 0–5 scores were 20.0%, 44.0%, 46.2%, 78.6%, 77.3%, and 92.3% respectively. As in the original article, the scores were used to define four risk groups: group A, score of 0; group B, score of 1–2; group C, score of 3–4; and group D, score of 5. A plot of sensitivity versus 1 – specificity (receiver operating characteristic [ROC] curve) and the area under the curve (AUC) were calculated.

## 3 RESULTS

**FIGURE 1:**
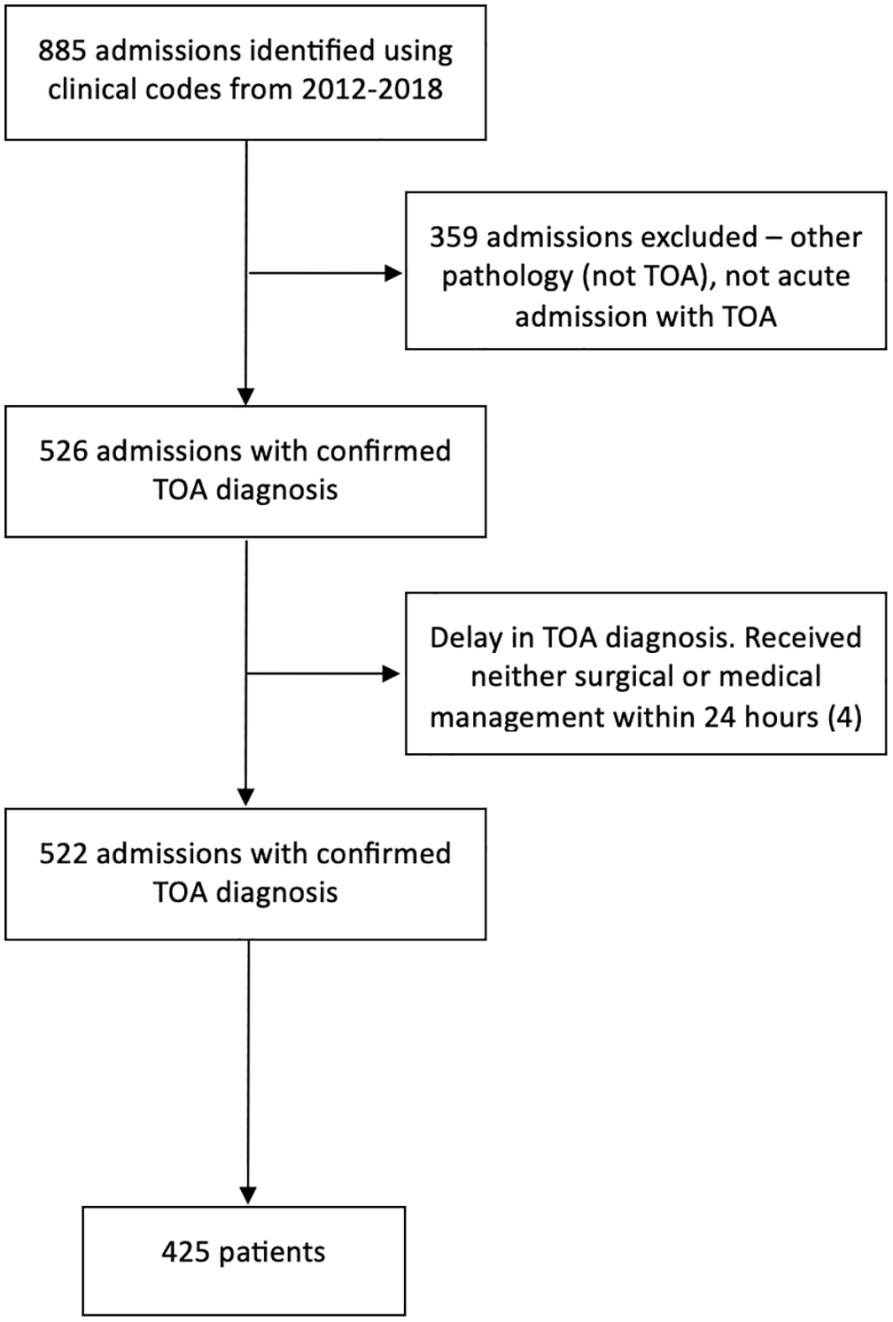
Study flowchart.

There were 522 admissions with TOA for 425 patients over the study period (Figure 1). Of these, 359/425 (84%) patients had one admission, 43/425 (10%) had two admissions, 16/425 (4%) had three admissions, 6/425 (1%) had four admissions, and 1/425 (<1%) was admitted five times with a new, recurrent, or persistent TOA.

Most admissions (504/522, 96.4%) had a diagnosis of TOA based on radiological findings, and most of those diagnosed by surgery were in the early intervention group (17/19, 89.5%). Reasons for diagnosis being made at time of surgery are as follows. For the early surgical intervention group, 11 admissions occurred under a general surgical specialty and surgery was performed without preoperative imaging. Four admissions had imaging that did not show TOA (one with an absence of pathology, and three suggesting an alternate pathology) but patients were found to have TOA at surgery. In two cases, TOA was the presumed diagnosis based on history and imaging consistent with TOA in a community scan or previous admission. For the initial medical intervention group, in two cases, TOA was the presumed diagnosis based on history and imaging consistent with TOA in a community scan or previous admission.

Baseline demographics and clinical characteristics are described in Table 1. Most patients (450/522, 86%,) were managed medically. In the 72/522 (14%) admissions who had early intervention, 68/72 (94%) were managed surgically. Only 4/72 (6%) patients had radiological drainage of TOA. Those admissions that had early intervention had larger abscesses and higher inflammatory markers (WCC, neutrophils, CRP) than those who had initial medical management, suggesting that more severe disease was present in that group.

**TABLE 1:** Demographic and admission features for patients with tubo‐ovarian abscess (TOA).

Age (years)	Early intervention intended							
	Surgical		Radiological		Medical treatment intended		Total	
	(*n* = 68)		(*n* = 4)		(*n* = 450)		(*n* = 522)	
	Median (IQR)		Median (IQR)		Median (IQR)		Median (IQR)	
	39 (29–45)		42 (26–52)		40 (31–46)		40 (31–46)	
	*n*	%	*n*	%	*n*	%	*n*	%
Ethnicity								
NZ European	8	11.8%	0	0.0%	66	14.7%	74	14.2%
Māori	13	19.1%	1	25.0%	119	26.4%	133	25.5%
Pacific Island	36	52.9%	2	50.0%	219	48.7%	257	49.2%
Asian	10	14.7%	1	25.0%	43	9.5%	54	10.3%
Other European or MELAA	1	1.5%	0	0.0%	3	0.7%	4	0.8%
Type of imaging^a^								
Ultrasound only	17	25.0%	0	0.0%	222	49.3%	240	46.0%
Ultrasound + other^b^	6	8.8%	2	50.0%	132	29.3%	141	27.0%
CT only	27	39.7%	2	50.0%	92	20.4%	121	23.2%
MRI +/− CT	1	1.5%	0	0.0%	2	0.4%	3	0.6%
No imaging^c^	17	25.0%	0	0.0%	2	0.4%	17	3.3%
Abscess characteristics								
Bilateral	17	25.0%	3	75.0%	105	23.3%	125	23.9%
Dimensions	Median (IQR)		Median (IQR)		Median (IQR)		Median (IQR)	
Largest diameter (mm)	77 (60–91)		121 (96–134)		69 (55–86)		70 (55–86)	
Mean diameter (mm)	58 (46–71)		87 (67–92)		54 (43–67)		55 (43–67)	
History‐based risk factors								
Diabetes mellitus	9	13.2%	2	50.0%	83	18.4%	94	18.0%
Previous PID or TOA	13	19.1%	1	25.0%	151	33.6%	165	31.6%
Previous STI	13	19.1%	0	0.0%	93	20.7%	106	20.3%
Vaginal/abdominal surgery								
No	63	92.6%	3	75.0%	428	95.1%	494	94.6%
<2 weeks prior	4	5.9%	0	0.0%	12	2.7%	16	3.1%
2–6 weeks prior	1	1.5%	1	25.0%	10	2.2%	12	2.3%
Intra‐uterine device	22	32.4%	0	0.0%	89	19.8%	111	21.3%
Admission microbiology								
High vaginal swab NAAT								
Positive for chlamydia, gonorrhea or trichomonas	8	11.8%	0	0.0%	34	7.6%	42	8.0%
Not performed	15	22.1%	2	50.0%	29	6.4%	46	8.8%
Vaginal swab								
Positive any growth	12	17.6%	1	25.0%	60	13.3%	73	14.0%
Not performed	16	23.5%	1	25.0%	39	8.7%	56	10.7%
Admission laboratory values	Median (IQR)		Median (IQR)		Median (IQR)		Median (IQR)	
Hemoglobin (g/L)	121 (106–130)		115 (99–120)		121 (108–131)		121 (108–131)	
WCC (× 10^9^/L)	14.8 (11.4–18.4)		16.4 (13.9–21.7)		14.5 (11.1–17.8)		14.6 (11.2–17.8)	
Neutrophil (× 10^9^/L)	12.3 (9.3–15.9)		12.7 (11.1–18.4)		11.7 (8.3–14.9)		11.8 (8.5–15.0)	
Platelets	300 (234–390)^d^		485 (435–603)		332 (274–417)^e^		328 (271–416)	
C‐reactive protein (mg/L)	179 (83–250)		216 (156–255)		108 (42–197)^f^		116 (49–210)	
Creatinine (μmol/L)	65 (58–79)		78 (68–104)		67 (60–78)^g^		67 (60–78)	

*Note*: Data shown are median (IQR) or *n* (%).

Abbreviations: CT, computerized tomography; IQR, interquartile range; MELAA, Middle Eastern, Latin American and African; NAAT, nucleic acid amplification test; STI, sexually transmitted infection; WCC, white blood cell count.

^a^ Imaging modality used to diagnose TOA. Includes imaging performed during or within 6 weeks prior to admission.

^b^ Ultrasound + other – indicates patients had both an ultrasound and at least one other form of imaging (magnetic resonance imaging or computed tomography scan) to confirm diagnosis of TOA.

^c^ Radiologic data unavailable for 19 patients, as TOA diagnosis for that admission is based on surgery findings.

^d^ Data missing for *n* = 1.

^e^ Data missing for *n* = 2.

^f^ Data missing for *n* = 1.

^g^ Data missing for *n* = 4.

Medical management was successful (i.e. IV antibiotics were the only treatment required to discharge) in 293/450 (65.1%) cases. However, further intervention prior to discharge was performed in 157/450 (34.9%) admissions initially managed with medical therapy alone. Further intervention was predominantly surgical (139/157, 88.5%), with few (18/157, 11.5%) cases undergoing radiology‐guided drainage.

The characteristics of TOA with successful and failed initial medical management are shown in Table 2. Compared with those where medical management was unsuccessful, those with successful medical management of TOA were less likely to be febrile on admission (33.1% vs. 49.7%), had TOAs that were smaller (mean largest diameter 65 mm [IQR 65–80] vs. 77 mm [IQR 63.0–93.0]), and had lower inflammatory markers on admission (CRP 89.5 mg/L [IQR 27–164.5] vs. 143.0 mg/L [78.0–250.0]); WCC 14.2 × 10^9^/L (IQR 10.9–17.1 vs. 15.2 × 10^9^/L [IRQ 11.9–18.4]).

**TABLE 2:** Admission features predictive of medical treatment failure in patients with tubo‐ovarian abscess (TOA).

	Successful medical (*n* = 293)		Failed medical (*n* = 157)		Unadjusted OR (95% CI)	*P*	Multivariable adjusted OR (95% CI)	*P*
Age (years)	40.0 (31.0–46.0)		40.0 (32.0–46.0)		1.00 (0.98–1.02)	0.732	1.00 (0.97–1.02)	0.642
Ethnicity								
Māori	68	23.2%	51	32.5%	1.17 (0.64–2.14)	0.088	0.98 (0.49–1.95)	0.158
Pacific Island	154	52.6%	65	41.4%	0.66 (0.37–1.15)		0.59 (0.31–1.10)	
Asian	29	9.9%	14	8.9%	0.75 (0.34–1.67)		0.83 (0.35–1.96)	
European/other	42	14.3%	27	17.2%	Ref		Ref	
Fever (≥37.5°C)	97	33.1%	78	49.7%	2.00 (1.34–2.97)	0.0006	1.72 (1.11–2.67)	0.016
Abscess characteristics								
Bilateral abscesses	62	21.2%	43	27.4%	1.41 (0.90–2.20)	0.137	1.50 (0.91–2.47)	0.111
Maximum diameter (mm)	65.0 (51.0–80.0)		77.0 (63.0–93.0)		1.02 (1.01–1.03)	<0.0001	1.02 (1.01–1.03)	0.0001
History‐based risk factors								
Diabetes mellitus	51	17.4%	32	20.4%	1.22 (0.74–1.99)	0.438	1.09 (0.61–1.93)	0.779
Prior PID, TOA	130	44.4%	69	43.9%	0.98 (0.67–1.45)	0.932	1.07 (0.69–1.67)	0.767
IUCD in situ	53	18.1%	36	22.6%	1.35 (0.84–2.17)	0.220	1.34 (0.78–2.28)	0.287
Admission laboratory values								
WCC (×10^9^/L)	14.2 (10.9–17.1)		15.2 (11.9–18.4)		1.04 (1.00–1.08)	0.035	1.00 (0.95–1.04)	0.824
CRP (mg/L)	89.5 (27.0–164.5)^a^		143.0 (78.0–250.0)		1.01 (1.00–1.01)	<0.0001	1.00 (1.00–1.01)	0.007
Creatinine (μmol/L)	67.0 (60.0–78.0)^b^		67.0 (60.0–78.0)		1.00 (0.99–1.00)	0.209	0.98 (0.99–1.00)	0.342

*Note*: Data shown are median (interquartile range) or *n* (%).

Abbreviations: CI, confidence interval; CRP, serum C‐reactive protein; creatinine, serum creatinine; IUCD, intrauterine contraceptive device; OR, odds ratio; PID, pelvic inflammatory disease; TOA, tubo‐ovarian abscess; WCC, white blood cell count.

^a^ Missing data, *n* = 1.

^b^ Missing data, *n* = 4.

The factors associated with failed medical management after multivariable analyses (Table 2) included presence of fever (temperature of 37.5°C or higher) on admission (1.72 odds of failed management if febrile on admission), larger mean TOA diameter (2% higher odds for every 1‐mm increase in abscess size) and higher CRP (1% higher odds for every unit increase in CRP) (1.00 [1.00–1.01]).

Laparoscopic surgical approach was favored in both early surgery and surgery occurring after failed medical management (Table 3). Those having surgery after failed medical management were less likely to have washout/drainage alone and more likely to have pelvic clearance. A composite of surgical morbidity/mortality was high, and similar for early or delayed surgery. Duration of stay and readmission rates within 6 weeks appeared to be higher in those with failed medical management; however, this should be interpreted cautiously due to the small numbers in each group.

**TABLE 3:** Surgical approach and type, complications, and readmission within 6 weeks.

	Early surgery (*n* = 68)		Surgery after failed medical management (*n* = 139)		Total surgically managed (*n* = 207)	
	*n*	%	*n*	%	*n*	%
Primary surgical approach						
Laparoscopy	35	51.5%	79	56.8%	114	55.1%
Laparotomy	33	48.5%	59	42.4%	92	44.4%
Posterior colpotomy	0	–	1	0.7%	1	0.5%
Surgery type						
Drainage/washout	53	77.9%	99	71.2%	152	73.4%
Adnexectomy	15	22.1%	29	20.9%	44	21.3%
Pelvic clearance	0	–	11	7.9%	11	5.3%
Complication within 6 weeks						
Wound complication^a^	5	7.4%	12	8.6%	17	8.2%
Thromboembolism	0	–	0	–	0	–
Visceral injury^b^	0	–	7	5.0%	7	3.4%
Death	1	1.5%	1	0.7%	2	1.0%
Composite (any of above)	6	8.8%	20	14.4%	26	12.6%

	Early surgical (*n* = 68)		Successful medical (*n* = 293)		Failed medical *n* = 157)		Total (*n* = 522)	
Length of stay (day) (median, IQR)	5.1 (4.0–6.7)		3.4 (2.5–4.7)		6.8 (5.2–8.5)		4.6 (3.0–6.6)	
Readmission within 6 weeks (*n*, %)	9	13.2%	53	18.1%	33	21.1%	96	18.3%

Abbreviation: IQR, interquartile range.

^a^ Includes wound infection requiring oral or intravenous antibiotics, or wound dehiscence.

^b^ Includes full‐thickness bowel or bladder wall injury.

**FIGURE 2:**
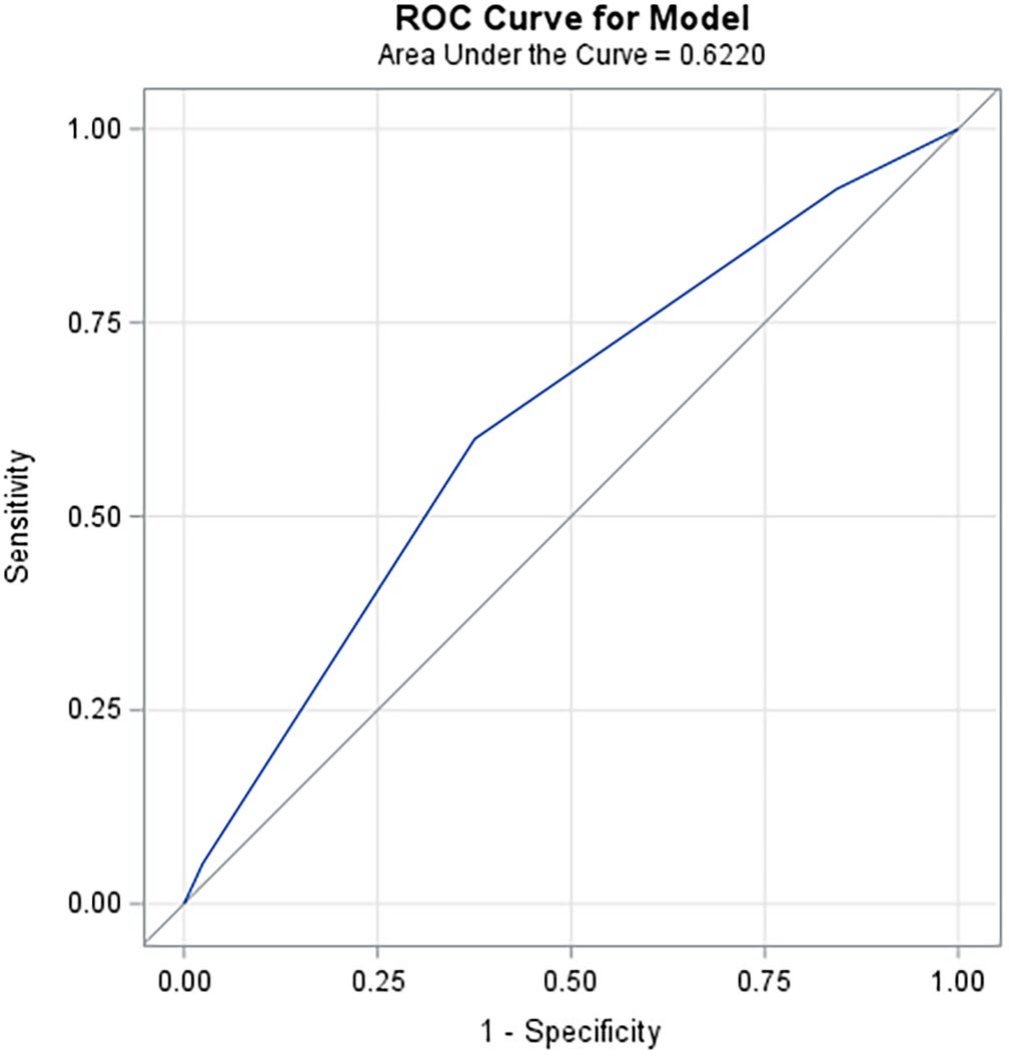
Receiver operating characteristic (ROC) curve. Curve demonstrates 1 – specificity (false positive rate) versus sensitivity for all cut‐off values measured from the test results of the cohort where initial intended treatment was medical (*n* = 450) presented in the manuscript.

The Fouks et al. prediction model was then applied to the group who initially were intended for medical management (*n* = 450), with a risk score for each admission using the four variables outlined above (Table 4; Figure 2). A plot of sensitivity versus 1 – specificity (ROC curve) and the AUC were calculated for the model described in Table 4 and is displayed in Figure 2. The area under the ROC curve was used to measure the discrimination of the prediction model. This was not as strongly predictive in our population as it was in the reference population used to develop the score, with an AUC of 0.62 (95% CI 0.57–0.67) compared with the AUC reported in the original study of 0.73 (95% CI 0.68–0.77).

**TABLE 4:** Risk score as a categorical predictor of medical management success versus failure. The risk assessment score as described by Fouks et al.^2^ = {[1 × age (>36 years)] + [1 × mean white cell count (≥16 × 1000/mm^3^)] + [2 × abscess diameter (≥70 mm)] + [1 × bilateral abscess]}. This gives a resulting score of 0–5. As in the original article, the scores were used to define four risk groups: Group A, score of 0; group B, score of 1–2; group C, score of 3–4; and group D, score of 5.

		Successful medical (*n* = 293)		Failed medical (*n* = 157)		Odds ratio (95% CI)	Model *P*‐value	AUC (95% CI)
		*n*	%	*n*	%			
Risk score								
Group A	0	46	15.7%	12	7.6%	Ref	<0.0001	0.62 (0.57–0.67)
Group B	1 or 2	137	46.8%	50	31.8%	1.40 (0.69–2.85)		
Group C	3 or 4	103	35.2%	85	54.1%	3.16 (1.58–6.35)		
Group D	5	7	2.4%	8	5.1%	4.38 (1.32–14.50)		
Missing		0	0.0%	2	1.3%			
Components that went into risk score^a^								
Age >36 years		176	60.1%	98	62.4%	1.10 (0.74–1.65)	0.626	
WCC ≥16×10^9^/L		100	34.1%	73	46.5%	1.68 (1.13–2.49)	0.010	
Bilateral abscess		62	21.2%	43	27.4%	1.41 (0.90–2.20)	0.137	
Max diameter ≥70 mm		123	42.0%	99	63.9%	2.44 (1.64–3.65)	<0.0001	

Abbreviations: AUC, area under the curve; CI, confidence interval; WCC, white cell count.

^a^ Age >36 years; WCC ≥16 × 10^9^/L and bilateral abscess all contribute 1 to the risk score, and max. diameter ≥70 contributes 2 to the risk score.

## 4 DISCUSSION

As antibiotics alone are often unsuccessful at treating TOAs, being able to predict who is most likely to benefit from additional therapies would be useful for triaging care. We identified that larger size of the abscess and higher inflammatory markers on presentation were predictive of requiring intervention beyond antibiotics. These factors should be considered in the clinical assessment when determining the severity of disease. However, these may not be the only aspects to consider, as, in our cohort, a risk score system based on these same characteristics did not discriminate between those who would require surgical treatment and those who could be treated with antibiotics alone. Across cohort studies, abscess size is the only consistent predictor of failed medical management.^2^, ^8^, ^9^, ^10^ There is no consensus either on whether abscess size alone should be a threshold for treatment or on what this threshold should be.^5^, ^8^, ^13^, ^15^, ^16^ Fever, abscess size and CRP levels were predictive of medical treatment failure in our study. All of these are direct markers for the severity of disease and are concordant with other smaller published cohorts (summarized in Table S1).^2^, ^7^, ^8^, ^9^, ^10^, ^11^, ^12^, ^13^, ^17^ Age, WCC, presence of diabetes, or bilaterality of abscess were not associated with treatment failure. The risk assessment score developed by Fouks et al., uses many of these factors, which may explain why the scoring system had poor discriminatory ability to predict medical treatment failure. Although our definitions of treatment failure were similar, the intervention rate in Fouks et al. was higher (49.8%) than ours (34.9%). In our service, there is a preference to take those who present as significantly unwell or with symptoms suggestive of abscess rupture for early surgery, while others are managed medically. Therefore, the most unwell patients were excluded from the validation aspect of this study, which may have altered the performance of the tool. As the true point of clinical equipoise is about management of the unwell but stable patient, we believe that limiting validation of the risk score tool for those where the intention was to treat medically was appropriate. Since publication of the risk score by Fouks et al., another score has been developed,^3^ whichi uses admission body temperature, CRP, and TOA size at imaging with a score of 0–3 for each component depending on the level. A total score of 4 or more was associated with a need for surgery.^3^ In our study, we were unable to collect the specific temperature of patients presenting with TOA and, therefore, were unable to calculate scores and validate this system within our cohort. As the components of their score were all factors found to be associated with failed medical management in our cohort, so it may be that this is a risk score better suited to our population.

Medical management of TOAs may be considered preferable to surgical, as it avoids the surgical and anesthetic risks associated with operating on a septic or unwell patient. Some specialists in our department have stated that a period of 24–48 h of antibiotic treatment prior to surgery may reduce inflammation and improve outcomes. There is no published evidence to support this, and this is not a recommendation contained in current guidance.^5^

Due to the retrospective nature of this study, we are unable to draw firm comparisons between outcomes of those who underwent early intervention and those who underwent intervention only after medical treatment failed. The observed duration of admission and proportion of those readmitted following discharge appear to be less for those who underwent planned early surgery. This is despite those having early surgery appearing to have more significant disease at the time of presentation. We believe that this emphasizes the importance of being able to accurately predict and triage those with TOA who will need surgery, and not deferring surgery in those who are likely to need it.

This cohort of patients with TOA is the largest published. We observed that TOA is diagnosed most in older women of reproductive age, and the proportion of cases who had concurrent chlamydia, gonorrhea, or an IUCD in situ was low. An unexpected observation was that most TOA presentations occurred in Pacific women. In New Zealand, Pacific persons experience higher rates of obesity.^18^ Others have observed that higher BMI was associated with TOA requiring surgery or drainage.^13^ There is an established association between soft tissue and surgical site infections and obesity, and obesity may influence outcome of infection through mechanisms including immune dysregulation, altered cell‐mediated immunity, as well as comorbidities associated with diabetes.^19^, ^20^, ^21^ Pacific persons in New Zealand experience higher levels of deprivation, poorer educational outcomes and lower income levels, which impact health and may provide additional barriers to early presentation and treatment. In our study, ethnicity was not independently associated with treatment failure, and further research into management and outcomes of TOA should collect ethnicity data, alongside BMI (or alternative measures of adiposity), and markers of socioeconomic dis/advantage in order to better understand the impact of these variables on the development of TOA.

A strength of this study is its large size. We used a broad range of codes to identify patients through clinical coding to capture all cases of TOA. As a single center, this may limit the generalizability of the results, and we are mindful that without internationally accepted guidelines, the decision to intervene will vary between clinicians and be influenced by patient factors not measured in this study. A limitation of this study is the definition of treatment success being tied to discharge from hospital. Longer term, more meaningful outcomes (such as long‐term recurrence, sub‐fertility, pain) are required to best inform clinical care. A literature review in 2009 looked at the pregnancy rates with different management approaches. In those without TOA rupture in that study, laparoscopic drainage was associated with higher pregnancy rates, ranging from 32% to 63%, compared with medical management alone, with reported pregnancy rates ranging from 4% to 15%.^22^

There is a need to prospectively collect data on management and outcomes of TOA, including long‐term impact on fertility and pain outcomes. As time to discharge and readmission rates may be improved with earlier intervention, we believe a clinical trial in this area is indicated. Our data in the context of other published literature suggest that abscess size alone is the most consistent predictor of medical treatment failure across different populations.

## 5 CONCLUSION

In our population, one‐third of TOAs managed medically required surgical intervention. Fever, higher inflammatory markers, and larger mass were predictive of requiring surgery. Further prospective studies are required to compare the outcomes of medical management alone with early intervention with drainage in those having risk factors predictive of treatment failure. Long‐term follow‐up should also be looked at, to support both short‐ and long‐term benefits from intervention.

## AUTHOR CONTRIBUTIONS

All authors contributed to the drafting of and have reviewed the final manuscript. AM: acquisition, analysis, interpretation of data; JW: conception, acquisition, interpretation of data; CH: acquisition, interpretation of data; LU: acquisition, interpretation of data; JW: analysis, interpretation of data; AVdM: conception, acquisition, interpretation of data; CO: conception, analysis, interpretation of data.

## CONFLICT OF INTEREST STATEMENT

The authors have no conflicts of interest.

## PRIOR PRESENTATION

This study was presented at the FIGO conference as an oral abstract in October 2023.

## Supporting information


Data S1:

## Data Availability

The data that support the findings of this study are available on request from the corresponding author. The data are not publicly available due to privacy or ethical restrictions.
