# Supplementary material for: The Tubo‐ovarian abscess study (TOAST): A single‐center retrospective review of predictors of failed medical management
Source: Int J Gynaecol Obstet. 2025 Mar 31;170(2):927–35. doi: 10.1002/ijgo.70100 (PMC12255917; doi:10.1002/ijgo.70100)
Supplement: Supplementary file 1 — Data S1: [file IJGO-170-927-s001.docx]

**Supplementary**

Supplementary table 1 – Summary of factors predictive of medical management failure in TOA from published cohort studies.

| **Author,**  **year published** | **Setting** | **Cohort size** | **Mean age**  **(years)** | **Medical failure** | **Factors significantly associated with medical management failure** |
| --- | --- | --- | --- | --- | --- |
| Marshall et al  2025 | South Auckland, New Zealand | 522 | 40 | 34.9% | Fever, CRP, larger mass |
| Farid et al 2016 | Boston,  USA | 113 | 40 | 24.6% | WCC, max diameter of TOA |
| Chan et al 2019 | Singapore | 136 | 38 | 18.4% | TOA >7.4 cm, BMI >24.9, fever |
| Fouks et al 2019 | Tel Aviv  Israel | 335 | 39 | 49.8% | Abscess diameter, older age, WCC and CRP, bilateral abscess |
| Levin et al 2019 | Jerusalem,  Israel | 91 | 39 | 42.8% | CRP, WCC, platelet count, Ca-125, largest diameter |
| Ribak et al 2020 | Kfar Saba,  Israel | 94 | 37 | 24.4% | Average TOA size, trend to CRP |
| Wong et al 2020 | Singapore | 102 | 39 | 14.7% | Age >40, TOA >7 cm, fever |
| Akselim et al 2021 | Bursa  Turkey | 146 | 38 (medical), 41(intervention) | 30.8% | Age, BMI, CRP, larger abscess |
| Yongue et al 2021 | Multicentre  West London  UK | 214 | 40 | 37% | Temperature, CRP, TOA diameter |
| Gözüküçük et al 2021 | Ankara  Turkey | 96 | 38.8 (medical), 43.7 (intervention) | 29.2% | Age, largest abscess (vol >40 cm^3^ or diameter >5 cm) |
| Jalloul et al 2022 | Houston  USA | 169 | 36 years | 49.8% | Age, diabetic, elevated WCC, fever, larger abscess size |
| Hwang et al 2022 | Incheon,  South Korea | 72 | 31.1 (medical), 47 (intervention) | 44% | Mean WCC, ESR, CRP, mass size |

- Farid H, Lau TC, Karmon AE, Styer AK. Clinical Characteristics Associated with Antibiotic Treatment Failure for Tuboovarian Abscesses. *Infect Dis Obstet Gynecol*. 2016;2016. doi:10.1155/2016/5120293
- Chan GMF, Fong YF, Ng KL. Tubo-Ovarian Abscesses: Epidemiology and Predictors for Failed Response to Medical Management in an Asian Population. *Infect Dis Obstet Gynecol*. 2019;2019. doi:10.1155/2019/4161394
- Fouks Y, Cohen A, Shapira U, Solomon N, Almog B, Levin I. Surgical Intervention in Patients with Tubo-Ovarian Abscess: Clinical Predictors and a Simple Risk Score. *J Minim Invasive Gynecol*. 2019;26(3):535-543. doi:10.1016/j.jmig.2018.06.013
- Levin G, Herzberg S, Dior UP, et al. The predictive role of CA-125 in the management of tubo-ovarian abscess. A retrospective study. *European Journal of Obstetrics and Gynecology and Reproductive Biology*. 2019;238:20-24. doi:10.1016/j.ejogrb.2019.05.004
- Ribak R, Schonman R, Sharvit M, Schreiber H, Raviv O, Klein Z. Can the Need for Invasive Intervention in Tubo-ovarian Abscess Be Predicted? The Implication of C-reactive Protein Measurements. *J Minim Invasive Gynecol*. 2020;27(2):541-547. doi:10.1016/j.jmig.2019.04.027
- Wong TTC, Lau HCQ, Tan TC. Retrospective study on the efficacy and prognostic factors of conservative versus drainage of tubo-ovarian abscesses. *Arch Gynecol Obstet*. 2020;302(3):679-683. doi:10.1007/s00404-020-05640-0
- Akselim B, Karaşin SS, Demirci A, Üstünyurt E. Can antibiotic treatment failure in tubo-ovarian abscess be predictable? *European Journal of Obstetrics and Gynecology and Reproductive Biology*. 2021;258:253-257. doi:10.1016/j.ejogrb.2021.01.011
- Yongue G, Mollier J, Anin S, et al. Tubo-ovarian abscess: A proposed new scoring system to guide clinical management. *International Journal of Gynecology and Obstetrics*. 2021;157(3):588-597. doi:10.1002/ijgo.13932
- Gözüküçük M, Yıldız EG. Is it possible to estimate the need for surgical management in patients with a tubo-ovarian abscess at admission? A retrospective long-term analysis. *Gynecol Surg*. 2021;18(1). doi:10.1186/s10397-021-01095-6
- Jalloul RJ, Thomas M, Ward C, Pedroza C. Clinical Predictors of Failed Medical Treatment in Patients with Tubo-ovarian Abscess: External Validation of a Recently Published Risk Score. *J Minim Invasive Gynecol*. Published online January 2022. doi:10.1016/j.jmig.2022.01.004
- Hwang JH, Kim BW, Kim SR, Kim JH. The prediction of surgical intervention in patients with tubo-ovarian abscess. *J Obstet Gynaecol (Lahore)*. 2022;42(1):97-102. doi:10.1080/01443615.2020.1867965
